# Topological data analysis of *Escherichia coli* O157:H7 and non-O157 survival in soils

**DOI:** 10.3389/fcimb.2014.00122

**Published:** 2014-09-05

**Authors:** Abasiofiok M. Ibekwe, Jincai Ma, David E. Crowley, Ching-Hong Yang, Alexis M. Johnson, Tanya C. Petrossian, Pek Y. Lum

**Affiliations:** ^1^Agricultural Research Service-US Salinity Laboratory, United States Department of AgricultureRiverside, CA, USA; ^2^Department of Environmental Sciences, University of CaliforniaRiverside, CA, USA; ^3^Department of Biological Sciences, University of WisconsinMilwaukee, WI, USA; ^4^Ayasdi, Inc.Menlo Park, CA, USA

**Keywords:** Shiga toxin, contamination, survival time, fresh produce, organic, conventional

## Abstract

Shiga toxin-producing *E. coli* O157:H7 and non-O157 have been implicated in many foodborne illnesses caused by the consumption of contaminated fresh produce. However, data on their persistence in soils are limited due to the complexity in datasets generated from different environmental variables and bacterial taxa. There is a continuing need to distinguish the various environmental variables and different bacterial groups to understand the relationships among these factors and the pathogen survival. Using an approach called Topological Data Analysis (TDA); we reconstructed the relationship structure of *E. coli* O157 and non-O157 survival in 32 soils (16 organic and 16 conventionally managed soils) from California (CA) and Arizona (AZ) with a multi-resolution output. In our study, we took a community approach based on total soil microbiome to study community level survival and examining the network of the community as a whole and the relationship between its topology and biological processes. TDA produces a geometric representation of complex data sets. Network analysis showed that Shiga toxin negative strain *E. coli* O157:H7 4554 survived significantly longer in comparison to *E. coli* O157:H7 EDL 933, while the survival time of *E. coli* O157:NM was comparable to that of *E. coli* O157:H7 EDL 933 in all of the tested soils. Two non-O157 strains, *E. coli* O26:H11 and *E. coli* O103:H2 survived much longer than *E. coli* O91:H21 and the three strains of *E. coli* O157. We show that there are complex interactions between *E. coli* strain survival, microbial community structures, and soil parameters.

## Introduction

Food-borne outbreaks associated with contaminated produce have heightened concerns about the adequacy of control measures for the safe production of fresh fruits and vegetables. In the past decade, there have been over 70 fresh produce-related outbreaks in the United States, and the risk and burden is continuous (Brandl, 2006; Lynch et al., 2009). These vegetables have been implicated in approximately 20 outbreaks resulting in approximately 700 illnesses and 20 deaths between 1996 and 2006 (Doyle and Erickson, 2008; Allerberger and Sessitsch, 2009). Although there are leafy green vegetable associated outbreaks caused by *Salmonella* and *Cyclospora*, a majority of them have been due to food contamination with *Escherichia coli* O157:H7 (Sivapalasingam et al., 2004). The most likely mechanisms of *E. coli* O157: H7 contaminations include contamination from soil amendments (i.e., manure, compost and compost teas), water (irrigation or flooding/runoff from adjacent land), wildlife, and airborne deposition from off-farm activities such as cattle/dairy and manure/composting operations (Franz et al., 2008, 2011; Fremaux et al., 2008; van Elsas et al., 2011). One of the worst incidents to date was a multistate *Escherichia coli* O157:H7 outbreak in August and September 2006, which was associated with consumption of fresh, bagged spinach that was traced to a field in California (California Food Emergency Response Team, 2007a,b; Cooley et al., 2007; Jay et al., 2007). During this outbreak, the CDC reported over 200 illnesses, 104 hospitalizations and 3 deaths.

Although *E. coli* O157:H7 is reported to be the predominant STEC serotype in the United States, more than 200 non-O157 STEC serotypes have been identified in animals or foods (Karch et al., 2005). Approximately, 60 of these serotypes have been incriminated in human diseases. Recent epidemiological studies have recognized additional non-O157 serotypes, including O26, O45, O91, O103, O104, O111, O113, O121, and O145, among STEC strains that were linked to severe human disease in the United States, Europe and parts of Latin America (Brooks et al., 2005; Caprioli et al., 2005; Bettelheim, 2007; Mathusa et al., 2010; Beutin and Martin, 2012).

The mechanisms by which the pathogen is introduced into the produce are not fully understood; however, it is hypothesized that plants become contaminated when grown in fields fertilized with improperly treated manure (Beuchat, 1999) or flood irrigation with water contaminated with cattle feces or contact with contaminated surface runoff (Hillborn et al., 1999; Ibekwe et al., 2004). Depending on the soil properties and environmental factors, the survival time of *E. coli* O157:H7 in soils varies from 1 week to 6 months, and even longer in some extreme cases (Maule, 2000; Mubiru et al., 2000; Jiang et al., 2002; Ibekwe et al., 2007, 2011; Franz et al., 2008; Semenov et al., 2008; Ibekwe and Ma, 2011; Ma et al., 2011).

In this study, we integrated environmental data with microbial community to assess relationships among these factors and the pathogen survival. To this end, we propose a systematic evaluation of the relative effectiveness of current and potential new intervention strategies to reduce or prevent contamination of produce by employing a new analysis method called topological data analysis (TDA) (Carlsson, 2009; Lum et al., 2013), to uncover environmental variables that are correlated with survival of *E. coli* O157. TDA is based on an area of mathematics called topology and its implementation allows topological techniques to be used to discover subtle signals or “shape” in complex data such as this dataset. This approach has been used in the past to discover hard-to-identify signal in other complex datasets around viral evolution, breast cancer, diabetes and effects on the metagenome due to environmental stress (Nicolau et al., 2011; Chan et al., 2013; Probst et al., 2014; Sarikonda et al., 2014). We used TDA to reconstruct the relationship structure of *E. coli* O157:H7 and non-O157 survival in 32 soils (16 organic, 16 conventional) from California (CA) and Arizona (AZ) with a multi-resolution output. We show that differential survivability of various *E. coli* strains are dependent on microbial community structures and soil parameters.

## Materials and methods

### Datasets and bacterial strains for the dataset

Environmental and metagenomic data were obtained from three studies of the survival pattern of *E. coli* O157:H7 and non-O157 from produce growing region of California and Arizona. The first study (Ma et al., 2012) examined the effects of environmental variables on the survival of *E. coli* O157:H7 EDL 933. The second study (Ma et al., 2013) examined the effects of 454 FLX-derived sequences from the same soils on survival of *E. coli* O157:H7 EDL933. The third study (Ma et al., 2014) examined the effects of environmental variables on the survival of *E. coli* O157:H7 and non-O157. All of the *E. coli* strains used in this study are described in Table 1. All soil properties are as reported by Ma et al. (2012).

**Table 1:** ***E. coli* O157 and non-O157 strains used for the study**.

Strain^*^	**Source**	**stx_1_**	**stx_2_**	**eae**	**hylA**	**References**
*E. coli* O26:H11	cow, Ontario, Canada	−	+	+	+	Louie et al., 1998
*E. coli* O103:H2	cow, Ontario, Canada	+	+	+	+	Louie et al., 1998
*E. coli* O91:H21	Human, OH, USA	+	−	−	+	Ito et al., 1990
*E. coli* O157 NM	–, AL, USA	+	+	+	+	Fields et al., 1997
*E. coli* O157:H7 4554	cow, Japan	−	−	+	+	Feng et al., 2001
*E. coli* O157:H7 EDL933	human, USA	+	+	+	+	Perna et al., 2001

*stx_1_, a gene coding for Shiga toxin1, stx_2_, a gene coding for Shiga toxin2, eae, a gene coding for intimin, and hylA, a gene coding for hemolysin. “+” and “−” indicate a gene was identified and not identified in a given E. coli strain, respectively. E. coli O157:H7 4554 is therefore stx negative*.

*−Indicates the source was not identified*.

* *Adapted from Ma et al. (2014)*.

### Collection, characterization, inoculation of soils samples, and survival

Soil samples were collected from three major fresh produce growing areas: Salinas Valley California, Imperial Valley, southern California, and Yuma, Arizona (Ma et al., 2012). *E. coli* O157:H7 culture, a 1.0 ml aliquot was transferred into a 250 ml flask containing 100 ml LB (Luria-Bertani) broth, and incubated at 37°C for 18 h to achieve early stationary phase. The cells were harvested by centrifugation at 3500 g (Beckman, Brea, CA), washed three times using 10 mM phosphate buffer (10 mM, pH 7.2), and finally resuspended in deionized water, and cells were added in soils to a final density of about 0.5 × 10^7^ CFU per gram soil dry weight (gdw^−1^) according to a method slightly adapted from Franz et al. (2008). About 500 g of the inoculated soil was transferred to a plastic bag which was closed but which had some holes at the top to allow air exchange for survival studies. The inoculated soils were sampled (1 g) at days 0, 3, 6, 10, 14, 20, 27, 34, 40, and 48 to determine the survival of *E. coli* O157 and non-O157 over time. Details of the experimental procedure had previously been described (Ma et al., 2012).

### Soil DNA extraction, pyrosequencing and sequence data analysis

Community DNA was extracted from 32 leafy green-producing soils using a Power Soil Extraction Kit (MO BIO Laboratories, CA) with the bead-beating protocol supplied by the manufacturer. The quality and concentration of the soil DNA were assessed using a NanoDrop ND-1000 spectrophotometer (NanoDrop Technologies, DE). The overall size of the soil DNA was checked by running an aliquot of soil DNA on a 1.0% agarose gel. The soil DNA samples (15.0 μl) were then submitted to Research and Testing Laboratories (Lubbock, TX) for PCR optimization and pyrosequencing analysis. Bacterial tag-encoded FLX amplicon pyrosequencing were carried out as previously described (Acosta-Martinez et al., 2008; Acosta-Martínez et al., 2010). The 16S universal Eubacterial primers 530F (5′-GTG CCA GCM GCN GCG G) and 1100R (5′-GGG TTN CGN TCG TTG) were used for amplifying the ~600 bp region of 16S rRNA genes. Primer and PCR optimizations were done at the Research and Testing Laboratories (Lubbock, TX) according to the protocol described previously (Acosta-Martínez et al., 2010; Gontcharova et al., 2010; Nonnenmann et al., 2010). All FLX related procedures were performed following Genome Sequencer FLX System manufacturers instructions (Roche, NJ, USA). Bacterial pyrosequencing population data were further analyzed by performing multiple sequence alignment techniques using the dist.seqs function in MOTHUR, version 1.9.1 (Schloss et al., 2009).

### Data analysis

All data were analyzed using the Ayasdi software (http://www.ayasdi.com). The Ayasdi software uses TDA as a framework for a large repertoire of statistical and machine learning methods. The description of the implementation of TDA as a software is described in detail in the following publication (Lum et al., 2013). Briefly, the output consists of a topological network with nodes and edges, where nodes are collections of data points and an edge connects any two nodes that have one or more common data points. In this analysis, the mathematical functions (called “lenses” in the software) used are principal metric SVD 1 and 2. Principal metric SVD lenses are used when the distance metric used is non-Euclidean. Statistical test used to look at significance between sub-networks or groups is the non-parametric Kolmogorov-Smirnov test (KS score). Variables used in the analysis are the following: chemical (Sodium (Na), iron (Fe), potassium (K), electrical conductivity or salinity (EC), copper (Cu), assimilable organic carbon (AOC), total nitrogen (TN), calcium (Ca), Nickel (N), organic carbon (OC), microbial biomass carbon (MBC), sulfate (SO_4_), water holding capacity (WHC), magnesium (Mg), zinc (Zn), phosphate (PO4), molybdenum (Mo), physical (sand, clay, silt, and bulk density) and biological (time till detection for *E. coli* O157:H7 EDL933 [ttd(d)], time till detection for *E. coli* O157:H7 strain 4555 [ttd (d) O157-4554], time till detection for *E. coli* O157:H7 non-motile strain 4555 [ttd (d) O157NM], time till detection for *E. coli* O91 [ttd (d) O91], time till detection for *E. coli* O26 [ttd (d) O26], operation taxonomic units (OTUs), *Nitrospira*, diversity index (*H*′), *Proteobacteria, Alphaproteobacteria, Chloroflexi, Bacteroidetes, Acidobacteria, Actinobacteria, Gemmatimonadetes, Firmicutes, Verrucomicrobia, Deltaproteobacteria, Gammaproteobacteria, Planctomycetes, Betaproteobacteria*).

## Results

### Soil sample site similarities and management network

**Figure 1:**
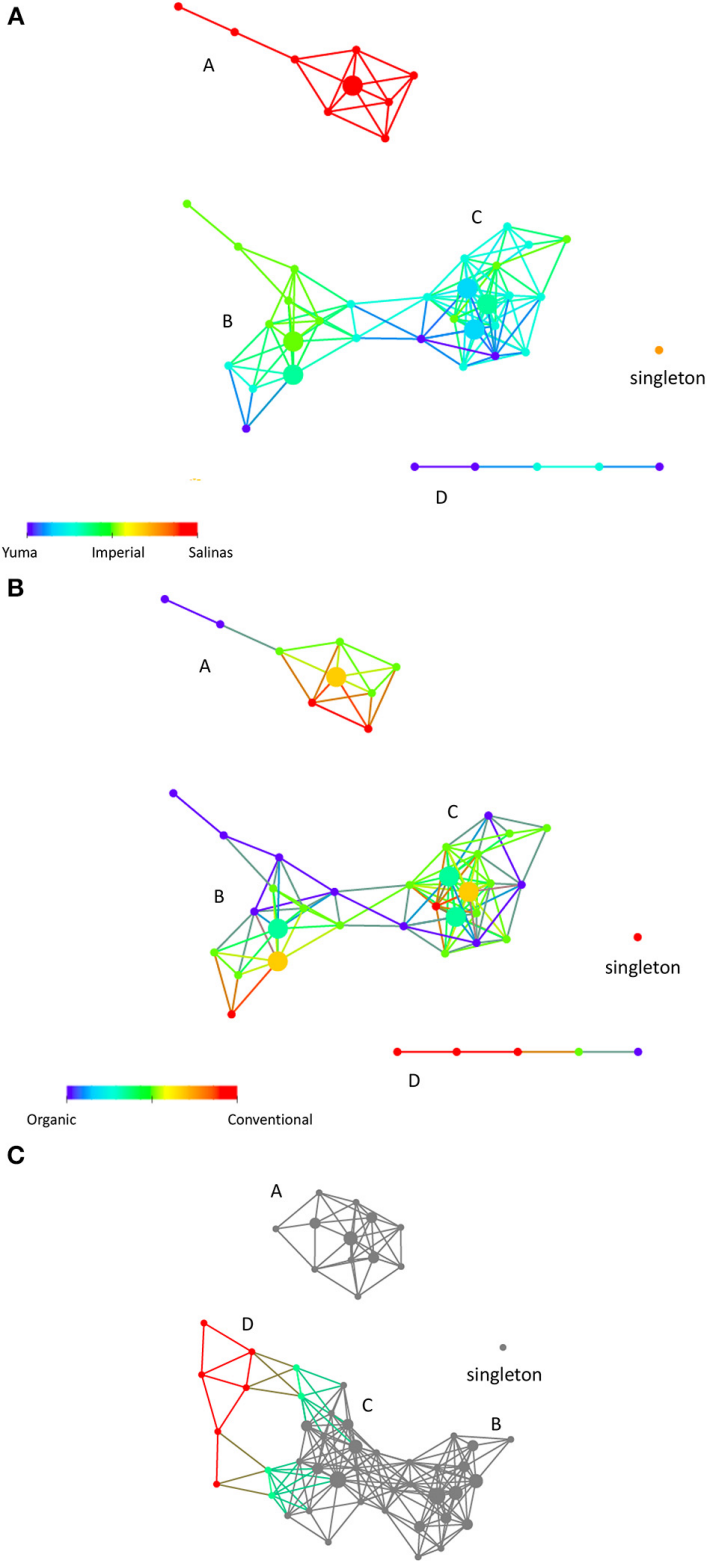
**(A)** Sample-sample relationships in a topological network. Using physical, chemical, and biological characteristics of the samples, we obtained a network that comprised of 4 sub-networks **(A–D)** and a singleton (single node comprising of 2 samples). The coloring here is by location, where each location is given a color (Salinas is red, Imperial Valley is green and Yuma is blue). Nodes that have a mixture of soils have colors in between as depicted in the color bar. Sub-networks structure indicates that physical, chemical, and biological characteristics primarily segregate the samples according to location, with Salinas being most different from soils from Yuma and Imperial Valley, **(B)**. The same network is colored by soilmanagement type (organic (represented by 0) vs. conventional (represented by 1). The red nodes represent samples with conventional soil management while the blue nodes represented the organic soil management. The green and orange colored nodes represented mixed organic and conventional soil management with varying percent of mixture of the two types of management. **(C)** Another network is built using the same parameters except for resolution. The soil samples are analyzed at a lower resolution to ask if structure **(D)** and the singleton will merge with any part of the sub-networks. Sub-network **(D)**, which comprised of samples from Yuma and Imperial, became part of sub-network **(B)** (colored nodes). Samples from sub-network **(D)** are not part of the gray nodes. The singleton however remained a singleton. The size of each node reflects the number of data points contained in the node. For **(A,B)**, the distance metric and filters were Person correlation and Principal Metric SVD and secondary metric SVD. Metric, Norm Correlation; Lens, Principal Metric SVD value (Resolution 30, Gain 4.0x, Equalized) Secondary Metric SVD Value (Resolution 30, Gain 4.0x, Equalized). For **(C)**, all analysis parameters remained the same except for resolution (20 instead of 30).

Using the properties of physical, chemical, and biological characteristic of these soil samples as variables, we clustered the soil samples using TDA. The resulting network represents the soil samples clustering into sub-networks. Figure 1A shows 4 sub-networks A, B, C and D with B and C connecting to form a larger sub- network. There is also a singleton (1 node comprising of 2 soil samples from Salinas that stood apart from everything else). The network can also be colored by various factors and characteristics such as location and soil management type for visualization (Figure 1). In addition, we can also apply statistics to probe what factors distinguished our soils into sub-networks. We found that “location” was one of the key differences between the sub-networks (Kolmogorov-Smirnov test *PV* < 0.0003). In order to visualize the effect of “location” on the soil samples, Figure 1 is colored by “location.” We show that soil samples from the Salinas areas (A) completely formed a separate sub-network from soil samples from the Imperial and Yuma areas (B, C, and D) as indicated by the color. This indicates that physical, chemical, and biological characteristic of these soil samples collectively are quite different from location to location, especially the soil samples from Salinas, which formed a distinct sub-network (A). Soil samples from Yuma and Imperial are closer to each, forming a sub-network that looks like a dumb bell, with some samples from Imperial clustering at left side of dumb bell (B) and the rest of the network comprised of a mixture between samples from Yuma and Imperial. Interestingly, physical, chemical, and biological properties measured of these soil samples did not differentiate between conventional and organic soil management as seen from the non-enrichment of any one type of soil management in the network (also see Table 2, where the *P*-value for soil management as a differentiating factor between those sub-networks was 0.4126). To further investigate sub-network D and the singleton, another network analysis was performed using the same distance metric and mathematical lenses but at a lower resolution (20 instead of 30). Sub-network D, which comprised of samples from Yuma and Imperial, became part of sub-network C (Figure 1C). The singleton however remained a singleton, indicating that these samples are fundamentally different from the rest of the samples due to unknown reasons including quality of the samples.

**Table 2:** **Kolmogorov-Smirnov test and *t*-test to identify soil and biological properties that best differentiate between Salinas Valley and Imperial/Yuma Valley locations**.

Column name^*^	**Signed KS-score**	**KS-score**	***t*-test *p*-value**
Clay	−0.8571	0.8571	0.0012
Na+	−1	1	1.05E–07
Fe	0.7321	0.7321	0.0299
ttd(d)	0.8125	0.8125	8.18E–05
K+	−0.5446	0.5446	0.0848
EC	−1	1	9.07E–06
*Nitrospira*	0.75	0.75	0.0014
Cu	−0.3660	0.3660	0.0607
tdd(d)_O1574554	−0.6666	0.6666	0.4823
Diversity index (*H*′)	0.7321	0.7321	0.0026
Molybdenum	−0.8125	0.8125	3.62E–04
*Proteobacteria*	−0.8125	0.8125	1.63E–04
WSOC	0.6696	0.6696	0.0259
T-N	0.375	0.375	0.1693
Ca++	−0.875	0.875	5.08E–04
Ni	0.4375	0.4375	0.1660
*Alphaproteobacteria*	0.6875	0.6875	0.0047
OC	−0.6517	0.6517	0.2614
MBC	−0.3839	0.3839	0.3520
SO4–	−0.9375	0.9375	4.55E–05
pH	−0.5714	0.5714	0.0259
*Chloroflexi*	0.5446	0.5446	0.03441
tdd(d)_O157NM	0.6666	0.6666	0.0995
Sand	0.7321	0.7321	0.0027
Bulk density	−0.5446	0.5446	0.0162
*Bacteroidetes*	0.7321	0.7321	0.0042
WHC	−0.5267	0.5267	0.2032
tdd(d)_O91	0.875	0.875	4.12E–06
*Acidobacteria*	1	1	0.0197
*Actinobacteria*	0.6071	0.6071	0.0240
Mg++	−0.7142	0.7142	0.0012
tdd(d)_O26	1	1	0.0796
*Gemmatimonadetes*	0.75	0.75	0.0012
Silt	−0.4821	0.4821	0.0403
*Firmicutes*	0.4821	0.4821	0.1989
*Verrucomicrobia*	0.4375	0.4375	0.3981
*Deltaproteobacteria*	0.9375	0.9375	8.55E–06
*Gammaproteobacteria*	−0.8125	0.8125	6.92E–05
Zn	−0.5892	0.5892	0.0056
*Planctomycetes*	0.8571	0.8571	0.0044
PO4—	1	1	0.0399
tdd(d)_O103	0.875	0.875	2.84E–06
*Betaproteobacteria*	0.75	0.75	1.95E–05
OTUs	−0.75	0.75	0.0015
Location	0.875	0.875	3.04E–06
Management	−0.258	0.258	0.4128

* *Meaning of abbreviations under column names: Na, Sodium; Fe, iron; ttd(d), time till detection for E. coli O157:H7 strain 933; K, potassium; EC, electrical conductivity; Cu, copper; ttd (d) O157–4554, time till detection for E. coli O157:H7 strain 4555; AOC, assimilable organic carbon; TN, total nitrogen; Ca, calcium; N, Nickel; OC, organic carbon; MBC, microbial biomass carbon; SO_4_, sulfate; ttd (d) O157NM, time till detection for E. coli O157:H7 non-motile strain 4555; WHC, water holding capacity; ttd (d) O91,time till detection for E. coli O91; Mg, magnesium; ttd (d) O26, time till detection for E. coli O26; Zn, zinc; PO_4_, phosphate; OTUs, operation taxonomic units. Signed KS score: the minus sign indicates that the attribute indicated in the column name is on average smaller in value in Salinas Valley compared to Imperial/Yuma Valley locations*.

**Figure 2:**
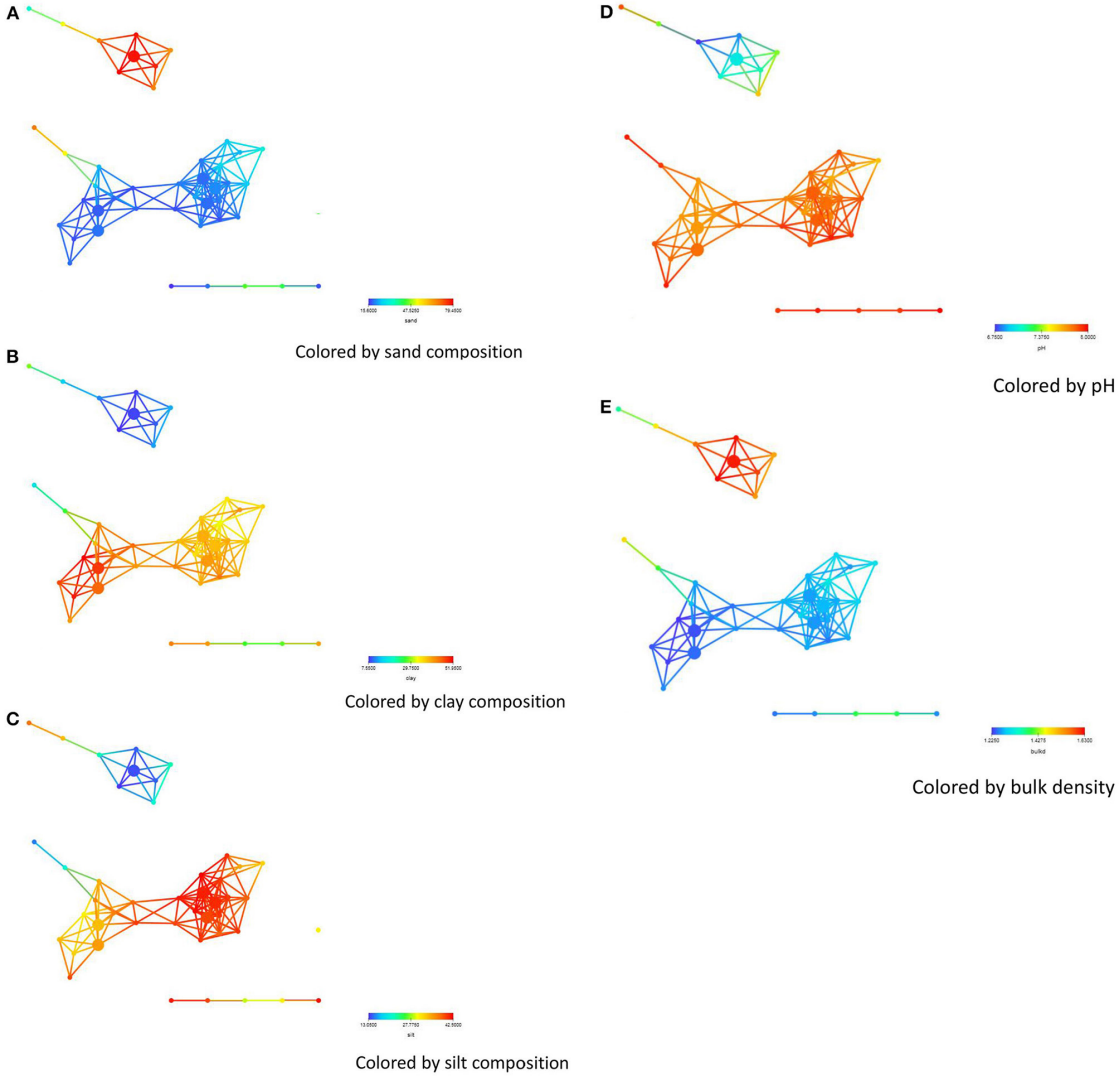
**Network colored by soil physical property concentrations-(A) sand, (B) clay, (C) silt, (D) pH, (E) bulk density**. The legend below the figures shows ranges in concentrations of some of the soil physical properties. The distance metric and filters are as shown in the legend of Figure 1.

Statistical analysis to identify key distinguishers of these sub-networks were performed on all numerical columns on all data points (Table 2) including detection times, biodiversity measures, management, location, sand, silk, clay, soil pH, bulk density, assimilable organic carbon (AOC), organic carbon (OC), microbial biomass carbon (MBC), electrical conductivity EC), chemical compound (Na^+^, K^+^, Ca^2+^, Mg^2+^ etc.) and bacterial phyla. Soil sand content was significantly higher (*P* = 0.0027) for soils from the Salinas Valley area (Figure 2A), whereas silt and clay contents were significantly higher (*P* = 0.0403 for silk and 0.0012 for clay) in soils from the Imperial and Yuma Valley areas (Figures 2B,C). Soil pH was between 6.7 and 8.0, with significantly higher pH (*P* = 0.025) occurring in the Yuma/Imperial Valley areas (Figure 2D). Soil bulk density values ranged between 1.22 and 1.63 mg, with soils from the Salinas Valley having significantly higher bulk densities (*P* = 0.0162; Figure 2E). Statistical tests indicated that total iron, PO_4_ and calcium were significantly higher (*P* = 0.0299; 0.0399; 5.08E−4, respectively) in Salinas Valley samples than samples from Yuma and Imperial Valleys. On the other hand, sodium and sulfate were significantly higher (1.05E−07; −05, respectively) in Yuma and Imperial Valley samples. No differences were observed among the locations in soil contents of total nitrogen (TN).

### Survival behavior of *E. coli* O157:H7 in soils

**Figure 3:**
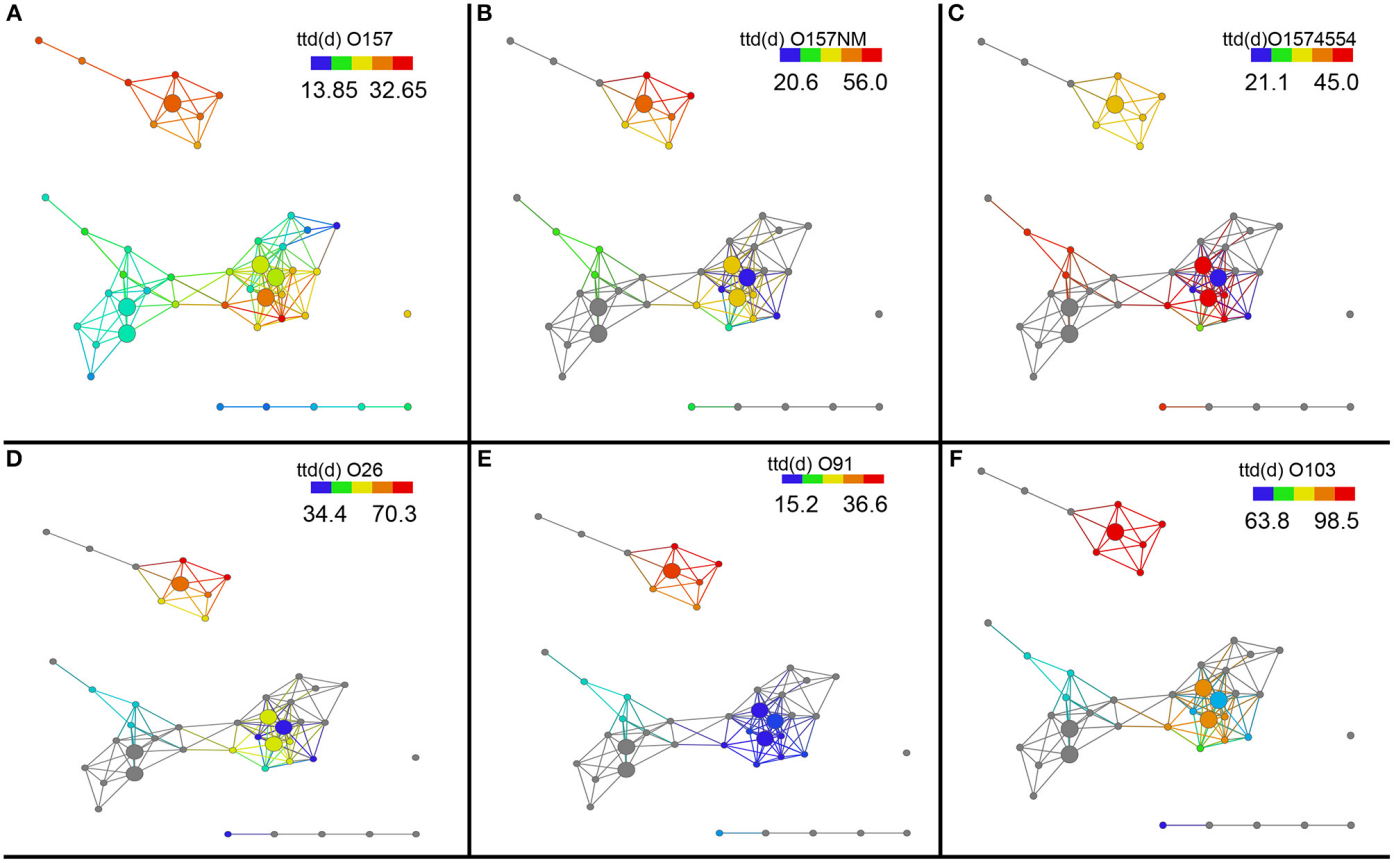
**Topological network data analysis of survival of *E. coli* O157:H7 and non-O157:H7 across the sub-networks identified in Figure 1A**. Survival of *E. coli* O157:H7 EDL933, *E. coli* O157:NM, and *E. coli* O157 strain 4554 (stx-) and shown in **(A–C)**. Survival of non-O157 strains, *E. coli* O26:H21, *E. coli* O103:H2, and *E. coli* O91:H21are shown in **(D–F)**. Gray nodes represent missing ttd (d) measurements for *E. coli* O157:NM, *E. coli* O157 strain 4554, *E. coli* O26:H21, *E. coli* O103:H2, and *E. coli* O91:H21. The distance metric and filters are as shown in Figure 1.

Next, we investigated survival of different *E. coli* strains in these different soil sub-networks. The network remains the same but we can now probe the network to see if any survival variables show any significant trends between these sub-networks. To do this we colored the same network by the length of survival of *E. coli* O157:H7 EDL933, *E. coli* O157:NM, and *E. coli* O157 strain 4554 (stx-) across the topological network to observe if differences exist in the soil networks. The shortest survival time (ttd) was observed for *E. coli* O157:H7 EDL933 (13.8–32.6 days) while the longest was observed for *E. coli* O157NM (20.6–56.0 days) and *E. coli* O157:H7 strain 4554 as intermediate at 21.1–45.0 days (Figures 3A–C). Figure 3 is colored by survival time of the indicated strain for all the soils. We also performed statistical test on the survival times and show that the survival time of *E. coli* O157:H7 EDL933 was significantly longer in soils from the Salinas Valley area (8.18E–05), whereas the survival time of *E. coli* O157:NM and the stx- *E. coli* O157:H7 strain 4554 were not significantly different in soils from the Salinas Valley area (0.0995 and 0.4823, respectively) and in soils from the Yuma and Imperial Valley region (Table 2). Furthermore, the coloring pattern indicates no differences in survival (*ttd*) between organic and conventional soils from Imperial Valley and Salinas. Survival time was much shorter in the organic soil than the conventional soils with *E. coli* O157:NM. This can be observed by the deep blue color (Figure 3B).

**Figure 4:**
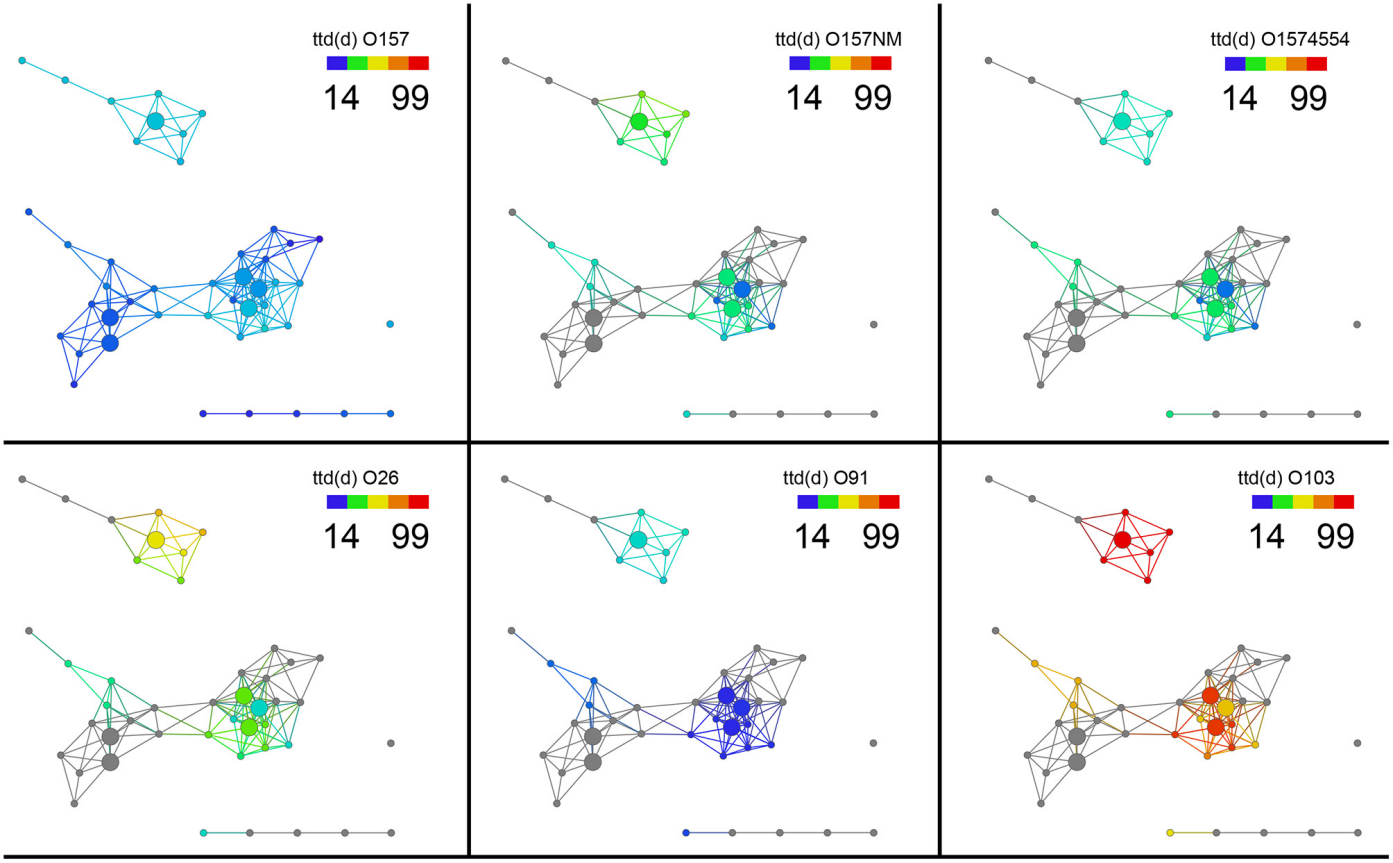
**Survival of six *E. coli* O157 and non-O157 strains grouped together on a normalized scale showed that *E. coli* O103:H2 survived the longest in all the soils, followed by *E. coli* O26:H21**. Gray nodes are as explained in Figure 3. The distance metric and filters are as shown in Figure 1.

Survival of non-O157 in soils was longer that *E. coli* O157:H7 except *E. coli* O91.H21. It was found that two non-O157 strains, *E. coli* O26:H21 and *E. coli* O103:H2 survived much longer that *E. coli* O91:H21. The three non-O157 strains survived significantly longer (*E. coli* O91.H21: 4.12E–06, O26:H21:0.079, and 0103: 2.84E–06) in soils from the Salinas Valley region than in soils from the Yuma and Imperial Valleys (Table 2). There were no differences of survival between organic and conventionally managed soils with the non-O157 strains. In the current study no isogenic strains (with and without stx) were used. When the six *E. coli* O157 and non-O157 strains were grouped together on the same scale it was shown that *E. coli* O103:H2 survived the longest in all the soils, followed by *E. coli* O26:H21 (Figure 4).

### Bacterial abundance and distribution as revealed by 454 pyrosequencing

**Figure 5:**
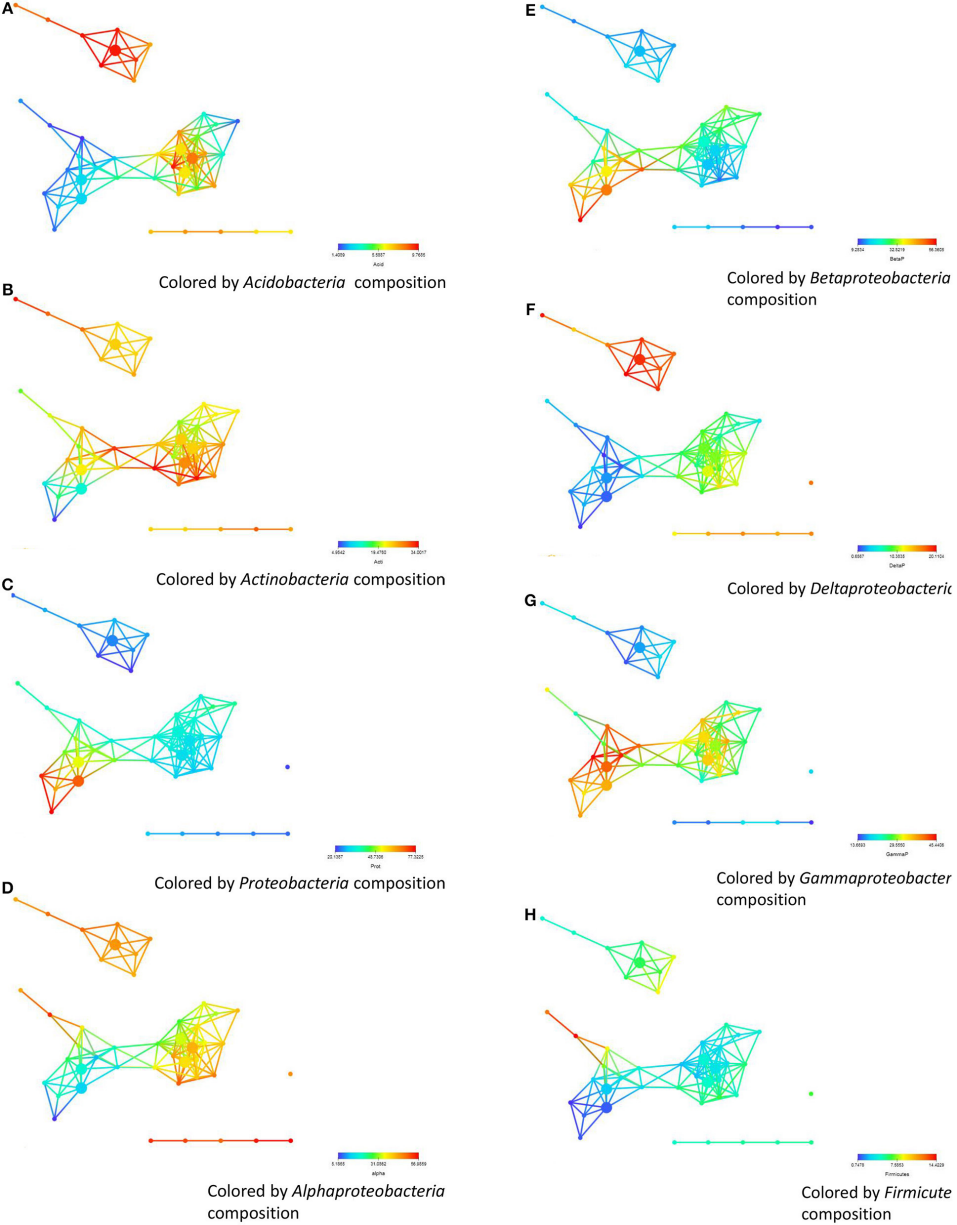
**The abundance and distribution of different bacterial phyla based on pyrosequencing and their correlation with survival. (A)** soils from Salinas Valley region, sub-networks **(B,C)** Yuma and the Imperial region. **(A–H)** represent the coloring of the abundance of *Actinobacteria, Acidobacteria, Firmicutes, Proteobacteria, Bacteroidetes, Alpha-, Beta-, Delta-*, and *Gammaproteobacteria*, respectively. The distance metric and filters are as shown in Figure 1.

We then analyzed how the abundance and distribution of different bacterial phyla based on pyrosequencing from the 32 soils collected from the three regions associated with the different regions clustered in the networks. By coloring the same networks with now the abundance of the different bacterial phyla, we show that there are marked differences between the distributions of the different bacterial phyla (Figure 5). As shown in Figure 5A the nodes that are colored red indicate significantly higher (*P* = 0.097) percentage of *Acidobacteria* (see Table 2 for details of the phyla) in soils from the Salinas Valley area than soils from Yuma and Imperial Valleys. These soils also contained a higher percentage of *Deltaproteobacteria* (*P* = 8.55E–06), *Alphaproteobacteria* (*P* = 0.0047) (Figures 5D,F) as seen by the color scheme. Significant differences in *beta* (*P* = 1.95E–05) and *Gammaproteobacteria* (*P* = 6.92E–05) were also observed in soils from Yuma/Imperial Valleys and soils collected from Salinas Valley area (Figures 5E,G). Significant differences were also found in *Proteobacteria* (*P* = 1.63E–04) (Figure 5C), *Actinobacteria* (*P* = 0.024) (Figure 5B), and no significant differences in *Firmicutes* (*P* = 0.1989) (Figure 5H) from the three regions. Further analysis showed that *Actinobacteria*, *Proteobacteria*, *Acidobacteria*, and *Bacteroidetes* were the dominant phyla among the bacterial communities in soils, and these four phyla accounted for about 75% of the total bacterial composition based on pyrosequencing (Figure S1). The current analysis has produced the same trends as the results obtained with our previous analysis that was based on correlation between survival time and dominant bacterial communities (Ma et al., 2013). In this earlier related study, stepwise multiple regression analysis was conducted and the results showed that EC, TN, and AOC were the most important factors impacting the survival of *E. coli* O157:H7 in all soils tested (Figure S2), with EC showing the most negative effect (*P* < 0.001) on survival and TN and AOC showing positive effects (*P* < 0.01) (Ma et al., 2012).

## Discussion

Analysis, interpretation, and visualization of complex data are major tasks confronting researchers today. Most data are presented in tabular formats after traditional statistical analysis. To better understanding the influence of selected soil properties and their impact on bacteria growth, we used TDA as implemented by the Ayasdi software. TDA can analyze disparate datasets in one setting, as well as presents topological networks as an informative visualization for understanding and interpretation. The TDA approach is sensitive to both large and small scale patterns that often fail to be detected by other analysis methods, such as principal component analysis, (PCA), multidimensional scaling, (MDS), and cluster analysis (Carlsson, 2009; Lum et al., 2013). In addition, we note that PCA and MDS produce 2-D scatterplots that are often hard to separate more subtle signal from noise. In addition, clustering methods produce distinct, unrelated groups that may obscure signal that is better captured using TDA, which is inherently suited to look for continuity in signal.

The three key concepts of topological analysis methods include coordinate freeness, which means that topology has the capability to measure properties of intrinsic shapes of data which is independent of the coordinate system. Coordinate free representations are vital when one is studying data collected with different technologies such as pyrosequencing or from survival data as we have used in this study or from different laboratories when the methodologies cannot be standardized (Lum et al., 2013). This is very critical to a study such as ours where the data collected are not from one uniform platform. As mentioned earlier, TDA has also been applied to various different studies to uncover complex signals (Nicolau et al., 2011; Chan et al., 2013; Lum et al., 2013; Romano et al., 2014; Sarikonda et al., 2014).

We have demonstrated that location is an important factor that we found to be associated with high survival of certain bacteria strains. Recent studies of metabolic network topologies across the bacterial tree of life revealed marked variation in network cluster and identified several genetic and environmental determinants affecting metabolic clustering (Parter et al., 2007). These authors showed that reduced metabolic cluster in single-species networks is associated with organisms inhabiting less variable environments. Our analysis, however, presents a unique characterization of microbial community-level cluster and demonstrates consistent differences that are associated with survival of *E. coli* O157:H7 from different locations. It should be noted that the correlation of certain bacterial phyla (*Actinobacteria* and *Acidobacteria*) with higher survival of *E. coli* O157:H7 does not necessarily mean causation of higher survival, and therefore, should be extrapolated very carefully. As discussed by Greenblum et al. (2012), *in silico* models of microbial communities are currently still scarce (Oberhardt et al., 2009) and mostly focus on simulated communities comprising a handful of species and on pair-wise interactions among community members (Stolyar et al., 2007; Freilich et al., 2010; Klitgord and Segrè, 2010; Wintermute and Silver, 2010). Experimental validation at the species or gene level of model components and parameters may be necessary for a successful and accurate understanding of individual species effects on survival. In essence, this study represents an important step in the development of a metagenomic systems biology approach. Such an approach can potentially advance metagenomic research in the same way systems biology advanced genomics, appreciating not only the parts list of a system but the complex interactions among parts and the impact of these interactions on function and dynamics.

In summary, the TDA networks identified various environmental factors that correlate with increased or decreased in survival of *E. coli* O157 in the three regions. In particular, we have identified a group of environmental factors such as EC, TN, AOC, etc. that consistently may enhance or inhibit survival of this pathogen from the three regions, and these factors were in agreement with some of our earlier studies from the same locations (Ma et al., 2012, 2013). We note that the effects of different environmental factors and bacterial community were easily detected by TDA because of the inherent ability of the analysis environment that allows analysis of all these factors simultaneously. Often times classical clustering approaches by themselves will miss these subtle signals because of the need to place data points into one cluster or another. This could end up highlighting only the most obvious signals while breaking up the more subtle ones.

As we move toward better understanding of how *E. coli* O157:H7 contamination could occur in the food chain, we believe a more holistic approach such as looking at all possible available factors together is important. However, because this creates complexity, there is a need to apply different approaches. We used here an approach to allow not only the mathematical analysis needed to uncover small signal but also the ability to visualize these complex relationships.

### Conflict of interest statement

The authors declare that the research was conducted in the absence of any commercial or financial relationships that could be construed as a potential conflict of interest.
