# Supplementary material for: Topological data analysis of Escherichia coli O157:H7 and non-O157 survival in soils
Source: Front Cell Infect Microbiol. 2014 Sep 5;4:122. doi: 10.3389/fcimb.2014.00122 (PMC4155871; doi:10.3389/fcimb.2014.00122)
Supplement: Supplementary file 1 [file Presentation1.PDF]

Supporting information

Topological Data Analysis of *Escherichia coli* O157:H7 and Non-O157 Survival in Soils

A. Mark Ibekwe[1,*], Jincai Ma[1,2], David E. Crowley[2], Ching-Hong Yang[3], Alexis M. Johnson[4], Tanya C. Petrossian[4], Pek Yee Lum[4]

1 USDA-ARS U. S. Salinity Laboratory, Riverside, CA 92507

2 Department of Environmental Sciences, University of California, Riverside, CA 92521

3 Department of Biological Sciences, University of Wisconsin, Milwaukee, WI 53211

4Ayasdi, Inc., Menlo Park, CA

Fig. S1. Abundance of dominated phyla revealed by 454-pyrosequencing targeting 16s rRNA genes. Different letters denotes significant differences (P = 0.05) among the three soils.

Fig. S2: Factorial biplot by the principal component 1 and 2 (PC1, 41.6%; PC2 26.0%) resulting from the principle component analysis (PCA) performed on soil properties and survival (*ttd*) of *E. coli* O157:EDL933 in soils. Salinity (EC, dS m^{-1}), water holding capacity (WHC, %), silt content (%), clay content (%), total nitrogen (TN, %); OC, organic carbon (%); AOC, assimilable organic carbon in soil water extract (mg kg^{-1}); MBC, microbial biomass carbon (mg kg^{-1}).

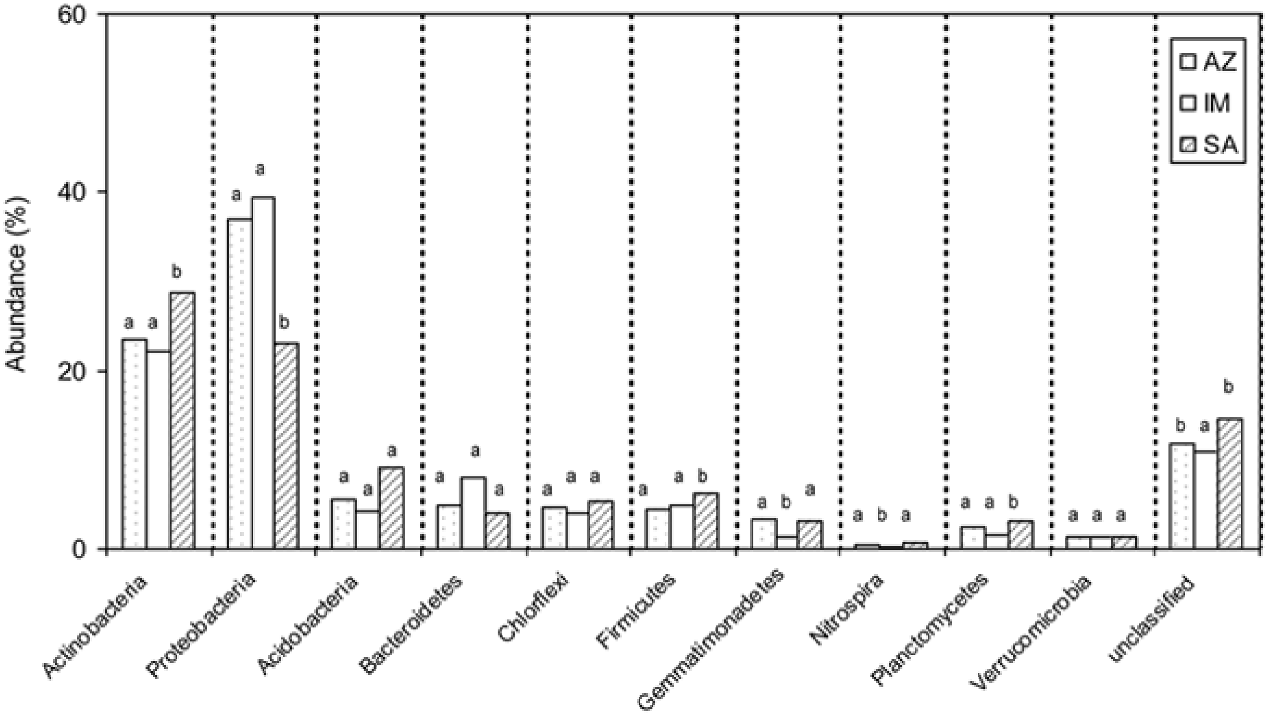

60
40
20
0
Abundance (%)
AZ
IM
SA
a
a
b
a
a
b
a
a
a
a
a
a
a
a
a
a
a
b
a
b
a
a
b
a
a
a
b
a
a
a
b
a
b
Actinobacteria
Proteobacteria
Acidobacteria
Bacteroidetes
Chloflexi
Firmicutes
Gemmatimonadetes
Nitrospira
Planctomycetes
Verrucomicrobia
unclassified

Fig. S1

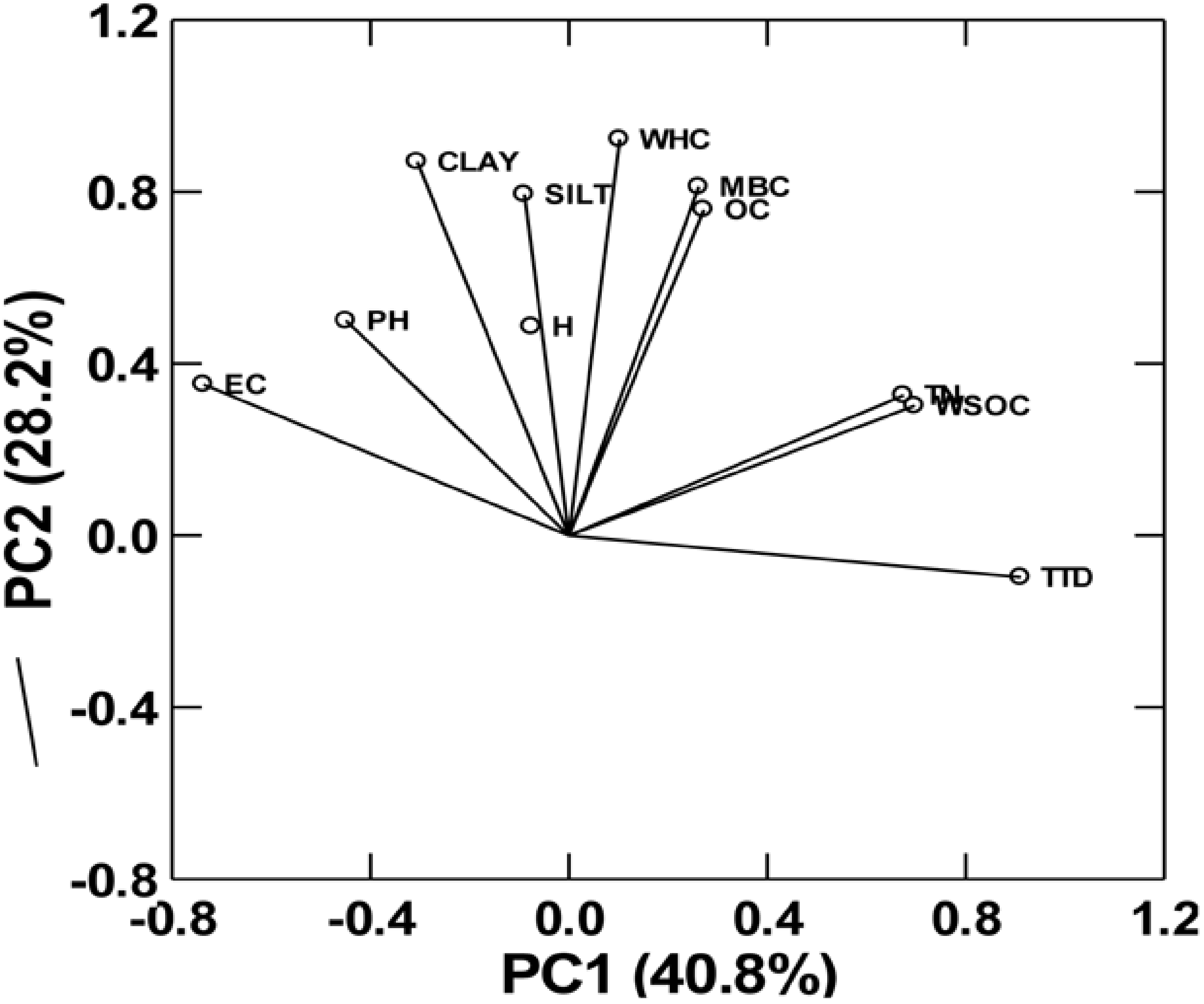

1.2
0.8
0.4
0.0
-0.4
-0.8
-0.8
-0.4
0.0
0.4
0.8
1.2
PC2 (28.2%)
PC1 (40.8%)
CLAY
WHC
SILT
MBC
OC
PH
H
EC
TN
WSOC
TTD

Fig. S2
